# Glucocerebrosidase mutations in primary parkinsonism

**DOI:** 10.1016/j.parkreldis.2014.09.003

**Published:** 2014-11

**Authors:** Rosanna Asselta, Valeria Rimoldi, Chiara Siri, Roberto Cilia, Ilaria Guella, Silvana Tesei, Giulia Soldà, Gianni Pezzoli, Stefano Duga, Stefano Goldwurm

**Affiliations:** a Dipartimento di Biotecnologie Mediche e Medicina Traslazionale, Università degli Studi di Milano, Milano, Italy; b Parkinson Institute, Istituti Clinici di Perfezionamento, Milan, Italy

**Keywords:** Parkinson's disease, *GBA*, Parkinsonism, Association analysis, Splicing mutation, Functional characterization

## Abstract

**Introduction:**

Mutations in the lysosomal glucocerebrosidase (*GBA*) gene increase the risk of Parkinson's Disease (PD). We determined the frequency and relative risk of major *GBA* mutations in a large series of Italian patients with primary parkinsonism.

**Methods:**

We studied 2766 unrelated consecutive patients with clinical diagnosis of primary degenerative parkinsonism (including 2350 PD), and 1111 controls. The entire cohort was screened for mutations in *GBA* exons 9 and 10, covering approximately 70% of mutations, including the two most frequent defects, p.N370S and p.L444P.

**Results:**

Four known mutations were identified in heterozygous state: 3 missense mutations (p.N370S, p.L444P, and p.D443N), and the splicing mutation IVS10+1G>T, which results in the in-frame exon-10 skipping. Molecular characterization of 2 additional rare variants, potentially interfering with splicing, suggested a neutral effect. *GBA* mutations were more frequent in PD (4.5%, RR = 7.2, CI = 3.3–15.3) and in Dementia with Lewy Bodies (DLB) (13.8%, RR = 21.9, CI = 6.8–70.7) than in controls (0.63%). but not in the other forms of parkinsonism such as Progressive Supranuclear Palsy (PSP, 2%), and Corticobasal Degeneration (CBD, 0%). Considering only the PD group, *GBA*-carriers were younger at onset (52 ± 10 *vs*. 57 ± 10 years, *P* < 0.0001) and were more likely to have a positive family history of PD (34% *vs.* 20%, *P* < 0.001).

**Conclusion:**

*GBA* dysfunction is relevant for synucleinopathies, such as PD and DLB, except for MSA, in which pathology involves oligodendrocytes, and the tauopathies PSP and CBD. The risk of developing DLB is three-fold higher than PD, suggesting a more aggressive phenotype.

## Highlights

• We screened a large case–control cohort with parkinsonism for common *GBA* mutations.
• *GBA* mutations in the Italian population are a risk factor for Lewy Bodies Diseases (PD and DLB).
• *GBA* mutations were not increased in the other forms of parkinsonism: PSP, CBD and MSA.
• *GBA* dysfunction does not seem to be involved in MSA and tauopathies.

## 1 Introduction

Mutations in the gene encoding beta-glucocerebrosidase (*GBA*, OMIM *606463) are an important and common risk factor for Parkinson's disease (PD). Many studies have shown an increased frequency of *GBA* mutations in PD compared to controls [1], [2]. In particular, an odds ratio (OR) of approximately 5 has been found in a multicenter study including approximately 5000 PD patients and an equal number of controls [3]. *GBA* mutations were repeatedly found to be increased also in Dementia with Lewy Bodies (DLB) [4], [5], [6]. A recent multicenter study on 700 DLB patients reported a remarkable OR of 8, suggesting that *GBA* mutations may have an even larger role in the genetic etiology of DLB than in PD [7]. Furthermore, PD carriers of the *GBA* mutation are more likely to progress to dementia, suggesting a significant impact on the distribution of pathology and on the resulting clinical phenotype [8]. On the other hand, Multiple System Atrophy (MSA) does not appear to be associated with *GBA* [9], [10]. So far, no data are available on *GBA* involvement in tauopathies, such as Progressive Supranuclear Palsy (PSP) and Corticobasal Degeneration (CBD).

The frequency and distribution of *GBA* mutations vary among populations, hindering comparisons between different patient series. Carrier frequency is quite high among Ashkenazi Jews (about 1 person in 14), and very rare in Asia [3]. In addition, studies were usually performed on series of patients with the same diagnosis often collected from many clinical centers, preventing the evaluation of the importance of *GBA* in the different forms of parkinsonism.

In this frame, we decided to analyze the major mutations in the *GBA* gene in a large series of unrelated patients with primary parkinsonism visited in a single Italian tertiary clinic and to compare their frequency with a large control group from the same population. This design enabled the evaluation and comparison of *GBA* burden in various forms of parkinsonism in the Italian population.

## 2 Methods

This study was approved by the local Ethical Committee and was conducted according to the Declaration of Helsinki and to the Italian legislation on sensitive personal data recording. Written informed consent was obtained from all subjects.

### 2.1 Subjects

We studied 2766 unrelated consecutive patients with degenerative parkinsonism, and 1111 controls, who contributed from 2002 to 2010 to the Parkinson Institute Biobank (www.parkinsonbiobank.com), regardless of family history or age at onset. All the patients had a diagnosis of primary degenerative parkinsonism: 2350 fulfilled current criteria for probable PD, 29 for DLB, 118 for MSA, 100 for PSP, and 34 for CBD [11], [12]. In particular, diagnosis of DLB was made only in those fulfilling the 1-year rule between the onset of dementia and parkinsonism [13]. In the remaining 135 cases, the clinical diagnosis was still uncertain and these patients are reported here as suffering from undefined primary parkinsonism (PKS). The majority of MSA cases (*N* = 113) had been previously reported in a multicentre collaborative study on *GBA* involvement in MSA [10]. Patients with suspect of secondary parkinsonism were excluded. All patients were examined by neurologists expert in movement disorders. The following clinical and demographic data were collected: gender, age at onset, asymmetry of symptoms at onset, disease duration, education, cigarette smoking, and family history of PD. Among the 2350 PD patients, the mean age at onset was 56.1 years (SD ± 10.9, range 13–87), the mean disease duration was 11.6 years (SD ± 6.7, range 5–56).

Controls were recruited among spouses and caregivers and were unrelated to the patients. All subjects who reported or showed signs or symptoms of movement disorders or other neurodegenerative diseases were excluded. Among the 1111 controls, the mean age at sample collection was 62.3 years (SD ± 11; range 30–94 years). All controls denied any family history for movement disorders in first-degree relatives.

Except for 25 patients originating mainly from other European countries, all patients and controls were of Caucasian ethnicity and Italian origin.

### 2.2 Mutation analysis

The mutational screening of *GBA* exons 9 and 10 was performed by a combination of high-resolution melting (HRM) analysis (exon 9) and direct DNA sequencing (exon 10). PCR primer couples were designed on the basis of the known genomic sequence of the gene (GenBank accession number NM_000157) to amplify the two exons of interest and their exon-intron boundaries, avoiding the concomitant amplification of the highly-homologous *GBA* pseudogene (*GBAP1*) (Supplementary Table 1). A detailed description of mutation analysis methods is reported in the Supplementary material.

The 2350 PD patients were previously tested for several PD-related genes, such as *LRRK2* (G2019S, R1441 C/G, I2020L), *Parkin*, *PINK1*, *DJ1*, and *SNCA* [14], [15], [16]. The 66 patients found to be carrier of mutations in these genes were not excluded from the *GBA* genetic analysis.

### 2.3 Molecular characterization of the newly-identified splicing mutation

The effect of the IVS10+1G>T splicing mutation on *GBA* pre-mRNA processing was evaluated in RNA derived from whole blood of the carrier patient. Whole blood was collected in a PAXgene Blood RNA Tube (PreAnalytiX, Hombrechtikon, Switzerland) and RNA purification performed by using the PAXgene Blood miRNA kit (PreAnalytiX) following the manufacturer's instructions. One microgram of total RNA was reverse transcribed (RT) using random nonamers and the Superscript-III Reverse Transcriptase (Invitrogen, Carlsbad, CA, USA). Of a total of 20 μL, 1 μL was used as template for standard PCR reactions by means of exonic primers (Supplementary Table 1). The identity of the amplified fragments was confirmed by Sanger sequencing. To quantify the relative amount of *GBA* exon-10 containing vs. skipping isoforms, we performed competitive RT-PCRs by using a 6-FAM-labeled primer. Amplified fragments were separated by capillary electrophoresis on an ABI-3130XL Genetic Analyzer (Applied Biosystems, Foster City, CA, USA) and quantitated by the GeneMapper v4.0 software.

### 2.4 Statistical analysis

All the following statistical procedures were performed using the R program release 2.8.0 (http://www.r-project.org/).

For each *GBA* mutation, standard case–control analyses on carrier frequency data were performed with the Fisher exact test; all *P* values are presented as non-corrected. The impact of *GBA* mutation burden on different clinical subtypes was evaluated by the use of the relative risk (RR) statistics.

The association of different variables with the presence of *GBA* mutations in PD patients was assessed by testing for differences between carriers and non-carriers by means of a chi-square test for categorical data (i.e. gender, asymmetric onset, smoking status, family history of PD), or by using the Student's *t* test for continuous data (i.e. age at onset, disease duration, education; these were analyzed as a quantitative variable, after having verified that their departure from linearity was not statistically significant). The effect of each factor was expressed as the OR and 95% confidence interval (CI). Unadjusted ORs were obtained by using a logistic regression model that included only the factor of interest; adjusted ORs were obtained by using a model that included the factor of interest plus all of the factors that were significant in the first step of analysis.

## 3 Results

### 3.1 Screening for *GBA* mutations on exons 9 and 10

In the whole cohort of subjects investigated (2766 patients and 1111 controls), we identified 10 different rare genetic variants, all present in the heterozygous state (Table 1).

**Table 1:** *GBA* mutations/rare variants identified in 2766 patients with degenerative parkinsonism and 1111 healthy controls.

Genomic position^a^	Rs ID	cDNA change^b^	Function^c^	Alleles in cases (*n*)	Alleles in controls (*n*)
1:155,205,659	NA	c.1225-24T>G	IVS8-24T>G	0	1
1:155,205,634	rs76763715	c.1226A>G	p.N370S^d^	69	4
1:155,205,581	rs149171124	c.1279G>A	p.E388K	5	1
1:155,205,440	NA	c.1388 + 32C>T	IVS9+32C>T	1	0
1:155,205,138	NA	c.1389-36C>G	IVS9–36C>G	0	1
1:155,205,107	NA	c.1389-5T>A	IVS9-5T>A	1	0
1:155,205,047	rs75671029	c.1444G>A	p.D443N^d^	1	0
1:155,205,043	rs421016	c.1448T>C	p.L444P^d^	47	3
1:155,204,985	NA	c.1505G>T	IVS10+1G>T^d^	1	0
1:155,204,978	rs371668537	c.1505C>A	IVS10+8C>A	1	0

All variants were identified in the heterozygous state. Frequent polymorphisms (minor allele frequencies >2.5%) are not reported.

Seventeen of the 47 L444P mutations found in cases and two of the 3 found in controls were associated with the p.A456P variant (indicating the presence of a complex recombinant allele).

Rs ID, refseq identification number; NA, not available [variant not reported either in the HGMD professional, or in 5400 exomes obtained from the Exome Variant Server, NHLBI GO exome Sequencing Project (ESP, v.0.0.9 data release, November 2011, http://evs.gs.washington.edu/EVS/)].

a According to UCSC Genome Browser (http://genome.ucsc.edu/, release Feb. 2009; GRCh37/hg19 assembly).

b According to mRNA Accession# NM_000157.3.

c Protein numbering omitting the signal peptide.

d Mutations already known to be responsible for Gaucher Disease.

The p.N370S and p.L444P mutations were more common in patients than in controls (2.5% *vs*. 0.36%, *P* = 1.2 × 10^−6^ OR = 7.1, CI = 2.6–19.5; and 1.7% *vs*. 0.27%, *P* = 1.1 × 10^−4^ OR = 6.4, CI = 2.0–20.6, respectively) (Supplementary Table 2). In addition to these two well-known major mutations, only the p.E388K variant was found in more than one subject, with no difference between cases and controls (0.18% vs. 0.09%, *P* = 0.68). Therefore we considered it a probable rare polymorphism. Indeed, E388K has never been clearly associated to Gaucher Disease (GD) [17], while has been found both in PD cases [5], [18] and controls [19], [20].

Other seven variants were found in individual cases (either patient or control). Among them, 2 were previously described as associated with GD: p.D443N and IVS10+1G>T [[21], HGMD professional, http://www.hgmd.org/], while the other 5 rare variants were found in intronic regions (Table 1).

### 3.2 Molecular characterization of the identified splicing variants

**Fig. 1:**
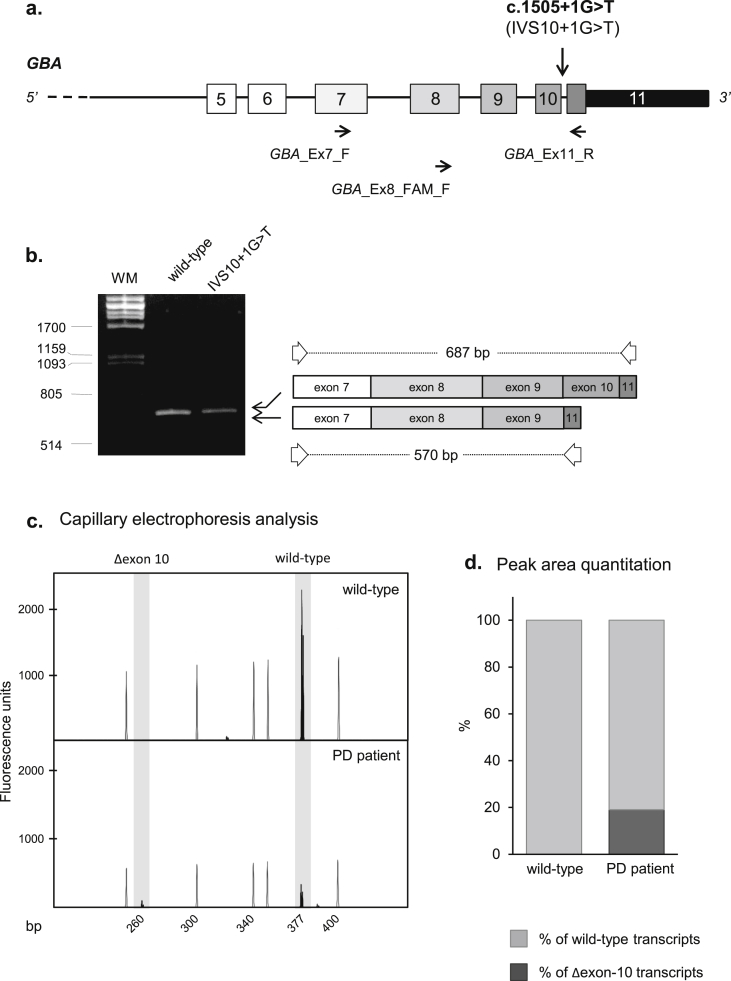
*In-vivo* analysis of the effect of the IVS10+1G>T mutation on *GBA* pre–mRNA splicing. a) A schematic representation of part of the *GBA* gene is reported: exons are represented by boxes (the thinner one corresponding to the 3′ untranslated region) and introns by lines. The gene is approximately drawn to scale. Arrows below the relevant exon indicate positions of primers used in RT-PCR experiments. The position of IVS10+1G>T is indicated by an asterisk. b) The results of RT-PCR experiments are shown. Amplified products were obtained from RNA extracted from whole blood of the PD patient carrying the IVS10+1G>T (c.1505+1G>T) mutation, as well as from a healthy donor. Products were separated on a 2% agarose gel; WM: molecular weight marker (λ*Pst*I). Two RT-PCR products were concomitantly amplified: one corresponding to the wild-type transcript, and a shorter one, of about 600 bp, possibly representing the transcript generated from the mutant allele. Direct sequencing of this aberrant product demonstrated that it is characterized by the in-frame skipping of the 117-nucleotide-long exon 10. A schematic representation of the two obtained RT-PCR products, together with their length, is also reported. c) RT-PCR experiments were also performed using, in the amplification step, a fluorescent primer. An aliquot of the fluorescent RT-PCR was run on a 3130XL Genetic Analyzer and measured with GeneMapper v4.0 software. The figure shows a GeneMapper window displaying fluorescence peaks corresponding to the 2 molecular species (wild type and mutant). The empty peaks represent the size standard (ROX-500 HD; their length is indicated below the panels), whereas the filled peaks correspond to the RT-PCR labeled products. The *X*-axis indicates GeneMapper data points and the *Y*-axis represents fluorescence units (FUs). d) Histograms indicate the quantitative analysis on fluorescence peak areas. For each sample, the sum of all the peak areas was set as 100%; the dark gray portion of the histogram corresponds to the percentage of transcripts containing transcripts lacking exon 10 (Δexon-10), whereas the light gray portion represents the percentage of wild-type isoforms. The expression of the mutant allele amounts to about one fourth of the wild-type allele.

Though previously described, the IVS10+1G>T splicing mutation has not yet been characterized from the molecular point of view. Hence, to evaluate the effect of IVS10+1G>T on *GBA* pre-mRNA processing, a cDNA region spanning exons 7–11 was amplified directly from mRNA of the carrier heterozygous patient. Results suggest that IVS10+1G>T is pathogenic through the skipping of exon 10 (Fig. 1a–b). The *GBA* transcript skipping exon 10 is predicted to code for a potentially non-functional protein, lacking 39 amino acids in the C-terminal portion of the enzyme.

The specific quantitative measurement of the mutant transcript showed that the expression of the mutant allele amounts to only about one fifth of the wild-type allele, suggesting that the aberrantly-spliced transcript might be unstable (Fig. 1c–d).

Concerning all the remaining 5 intronic variants (IVS8-24T>G, IVS9+32C>T, IVS9–36C>G, IVS9-5T>A, IVS10+8C>A), in-silico analyses did not predict any significant alteration of the splicing process (e.g. activation of a cryptic splice site or abrogation of a physiologic site; data not shown). Nonetheless, the IVS9-5T>A and IVS10+8C>A putative splice variants, both located within 10 nucleotides from intron-exon junctions, were expressed in HeLa cells. Analysis of transcripts generated from constructs containing either IVS9-5T>A or IVS10+8C>A showed that exon 10 is correctly included into the mature mRNA, demonstrating that both variants are neutral (Supplementary Fig. 1).

### 3.3 Frequency of *GBA* mutations

The frequency of *GBA* mutations according to clinical diagnosis is reported in Table 2.

**Table 2:** *GBA* mutation carriers according to clinical diagnosis.

Diagnosis	Subjects (*n*)	Carriers (*n*)	% Of subjects	RR (95% CI)	*P* value^a^
PD	2350	106	4.5%	**7.2 (3.3**–**15.3)**	**2.2 × 10**^**−11**^
DLB	29	4	13.8%	**21.9 (6.8**–**70.7)**	**9.9 × 10**^**−6**^
MSA	118	1	0.85%	1.3 (0.17–10.8)	0.56
PSP	100	2	2.0%	3.2 (0.67–15.1)	0.17
CBD	34	0	0%	2.1 (0.12–36.4)	0.99
Undefined PKS	135	5	3.7%	**5.9 (1.9**–**18.3)**	**5.9 × 10**^**−3**^
Healthy controls	1111	7	0.63%	/	/

All the pathogenic mutations (p.N370S, p.D443N, p.L444P, IVS10+1G>T) considered together. RR was calculated considering healthy controls as reference group.

Significant results are in bold.

Parkinson's disease (PD); Dementia with Lewy Bodies (DLB); Multiple System Atrophy (MSA); Progressive Supranuclear Palsy (PSP); Corticobasal Degeneration (CBD); Parkinsonisms (PKS).

a Statistics: Fisher exact test, two-tail.

Pathogenic *GBA* mutations (p.N370S, p.D443N, p.L444P, and IVS10+1G>T) were significantly increased in patients with PD, DLB, and undefined PKS, but not in those with MSA, PSP or CBD. The highest RR was in DLB (RR = 21.9, CI = 6.8–70.7, *P* = 9.9 × 10^−6^).

Five carriers (3.7%) occurred in the heterogeneous group of patients with an undefined PKS, containing all cases with a primary parkinsonism whose clinical features did not fulfill currently established consensus criteria [11], [12], [13]. All of them had parkinsonism (bradykinesia and rigidity) and co-occurring dementia, while three out of 5 had poor response to levodopa.

### 3.4 Genotype-phenotype correlations

We compared clinical phenotype between *GBA*-carriers and non-carriers in the PD subgroup only, given the relatively low number of carriers with other diagnoses (Table 3). We excluded from this analysis those patients who were carriers of mutations in other PD-related genes: 62 in the non-carrier group, and 4 in the *GBA*-carrier group (*LRRK2*-p.G2019S, *n* = 2; *LRRK2*-p.R1441C, *n* = 1; and homozygous deletion of exon 3 in parkin gene, *n* = 1). An extensive clinical description of these 4 PD patients is reported in the online Supplementary material.

**Table 3:** Cross-sectional analysis of demographic and general clinical features of 2284 consecutive patients with PD according to *GBA* mutation status.

Feature	*GBA* carriers (*n* = 102)	Non-carriers (*n* = 2182)	Unadjusted analysis		Adjusted analysis^a^	
			*P* value	Or (95% CI)	*P* value	Or (95% CI)
Male, *n* (%)	58 (56.9%)	1322 (60.6%)	0.45	1.17 (0.78–1.74)	–	–
Age at onset (*y*, mean ± SD)	51.54 ± 10.63	56.69 ± 10.51	**1.97 × 10**^**−6**^	**1.04 (1.03**–**1.06)**	**5.29 × 10**^**−6**^	**1.04 (1.03**–**1.07)**
Asymmetric onset, *n* (%)	78 (85.7%)^b^	1675 (85.1%)^b^	0.87	1.05 (0.58–1.91)	–	–
Disease duration (*y*, mean ± SD)	11.35 ± 7.315	11.87 ± 6.48	0.44	0.99 (0.96–1.02)	–	–
Education (*y*, mean ± SD)	10.70 ± 4.72^c^	9.80 ± 4.39^c^	**0.049**	**1.05 (1.00**–**1.09)**	0.31	1.02 (0.98–1.07)
Cigarette smoking, *n* (%)	39 (41.1%)^d^	797 (38.4%)^d^	0.60	1.12 (0.74–1.70)	–	–
Positive family history for PD, *n* (%)	21 (20.6%)	250 (11.6%)^e^	**7.4 × 10**^**−3**^	**1.97 (1.20**–**3.25)**	**0.019**	**1.87 (1.11**–**3.15)**

Values are expressed as counts (and %) or as mean ± standard deviation (SD). Significant values are in bold.

The age at which the patient noticed the first PD symptom was considered to be the age at onset of disease. Disease duration was calculated on the basis of the last examination. Education and smoking data are based on patient self-reporting. Current and former smokers were aggregated into the single category of smokers. The definition of positive family history was restricted to patients having at least one 1st degree relative with a formal diagnosis of PD.

a Variables were analyzed in a multivariate context through multivariate logistic regression (adjusting for all covariates that resulted significantly different between carriers and non-carriers in the “crude” analysis), with the presence of *GBA* mutations as dichotomous response variable.

b Percentage calculated on 91 carriers with available data and 1968 non-carriers.

c Value calculated on 98 carriers with available data and 2040 non-carriers.

d Percentage calculated on 95 carriers with available data and 2076 non-carriers.

e Percentage calculated on 2153 non-carriers with available data.

*GBA*-carriers had an earlier onset than non-carriers, with a mean age of 52 and 57 years, respectively. In particular, a total of 14/152 (9%) carriers had early onset (<40 years) compared to 88/2132 non-carriers (4%). Moreover, carriers were more likely to have a positive family history of PD (20.6% *vs* 11.6%).

## 4 Discussion

In this study we analyzed a large case series of patients consecutively collected at a single site, together with a large control group, to evaluate the impact of the most frequent *GBA* mutations in various forms of primary parkinsonism. *GBA* mutations contributed to PD and even more so to DLB, whereas they did not increase the risk for developing tauopathies (PSP and CBD).

Consistently with other European studies [3], [19], [20], the frequency of *GBA* mutations in PD among our Italian patients is 4.5%, confirming that *GBA* mutations are the most common genetic determinant of both familial and sporadic PD. However, in a previous study in the South of Italy, a lower frequency of mutations was found in PD patients (11/395, 2.8%) and in controls (1/483, 0.2%), and the most common mutation was p.L444P [22]. Conversely, we found that the most frequent genetic defect is p.N370S, as previously reported for Italian GD patients [23]. Our Institute is located in the North of Italy; however, it is a tertiary referral center and attending patients come from all parts of the country. Therefore, the difference may be due to a particular frequency of *GBA* mutations in Southern Italy or to the relatively small size of the case series analyzed by DeMarco and colleagues [22].

A limitation of our study is that we screened only exons 9 and 10, and therefore we lost the carriers of rare mutations in other regions of *GBA*. However, this screening strategy covers the vast majority of *GBA* mutations in our population [20], [23], and was chosen to be able to analyze a large number of individuals.

The p.L444P mutation, causing a more severe GD phenotype [23], does not appear to be associated with a greater risk compared to the less severe p.N370S (Supplementary Table 2), in contrast to what previously reported [24], [25]. This is relevant for genetic counseling. Considering the p.L444P and the recombinant p.L444P + p.A456P alleles separately, only the non-recombinant allele is significantly associated with a higher PD risk. However, this is probably due to the lower frequency of the p.L444P + p.A456P variant.

Moreover, our study provides a precise assessment of *GBA* mutation “carrier frequency” in the Italian population. Indeed, considering that the 2 major mutations account for around 70% of disease alleles, *GBA* mutation carriers including rare mutations should be 0.9% of the general population, consistently with a prevalence of GD in Italy of 1/40,000 [26].

Finally, we confirm that in *GBA*-carriers the disease onset occurs 5 years earlier than in non-carriers [3], at difference with what observed in our case series of *LRRK2*-mutated patients where age at onset was similar between carriers and non-carriers [16]. It remains to investigate the reason that why, although both genetic factors operate in a multifactorial context, their effect on the phenotype is expressed in a different way.

Considering other forms of parkinsonism, *GBA* mutations were most common in DLB, where the highest RR was found. Although our DLB cohort is too small to allow generalizations, this observation seems to confirm the trend towards more widespread pathological damage, even including the cortex and, consequently, the increased risk for dementia in *GBA* mutation carriers [1]. Consistently, the *GBA*-carriers amongst the patients categorized as undefined PKS had not only parkinsonism, but also dementia. This suggests that these patients are likely part of the Diseases with Lewy Bodies spectrum, a continuum of clinic-pathologic entities spanning from PD, PD and Dementia (PDD), to DLB [13]. In the future, it will be interesting to verify whether our *GBA* mutation carriers develop dementia more often than non-carriers, as suggested by other studies [3], [8].

Contrasting data on the frequency of *GBA* mutations in DLB compared to PD have been reported: lower for some authors [6], [18] and higher for others [4], [5]. In our analysis the frequency of *GBA* mutations was clearly higher in DLB than in PD, with an RR (21.9), which was considerably greater than in most studies. This may be due to the low number of cases with diagnosis of DLB in our study (note that the CI of the RR was 6.8–70.7), as well as to a selection bias at our Institute, which focuses on PD. Indeed, the DLB patients who have prominent dementia or dementia almost free from parkinsonism probably do not come to our attention. *GBA* may be more involved in forms with dementia with more important signs of parkinsonism. Future research to explore this hypothesis may focus on differences in cognitive and motor symptoms between PDD and LBD carriers. Furthermore it may be interesting to evaluate *GBA* mutation frequency within the LBD spectrum.

*GBA* mutations do not seem to play a role in the predisposition both to MSA, as previously evidenced by us [10] and others [9], and to tauopathies. This may underlie a different involvement of the *GBA*-mediated lysosomal impairment in different forms of parkinsonism. Indeed, in neurons, the connection between beta-glucocerebrosidase (GCase) impairment and Lewy bodies formation has been proposed to rely on GCase dysfunction leading to *α*-synuclein accumulation in the lysosome, which in turns exacerbates loss of GCase activity [27]. Unlike other synucleinopathies, the histopathologic hallmark of MSA is accumulation of *α*-synuclein within glial cytoplasmic inclusions (GCI) instead of within neurons. At the beginning of the pathogenic process, *α*-synuclein accumulates in the GCIs, located mainly within the oligodendroglial cells, leading to neurodegeneration and, ultimately, neuronal death [28]. The evidence that *GBA* mutations are not associated with the risk of developing MSA is in line with the hypothesis that the GCase impairment does not play an important role in oligodendrocytes.

Similar conclusions can be drawn for PSP and CBD tauopathies, in which to our knowledge *GBA* mutations have been sought for the first time. Indeed, although the mechanisms that lead to deposit of tau protein are unclear [29], the *GBA*-mediated lysosomal impairment does not seem to have a major role. This is of considerable interest, in view of the close correlation among *α*-synuclein, tau and amyloid *β* pathologies [30].
