# Supplementary material for: Glucocerebrosidase mutations in primary parkinsonism
Source: Parkinsonism Relat Disord. 2014 Nov;20(11):1215–20. doi: 10.1016/j.parkreldis.2014.09.003 (PMC4228056; doi:10.1016/j.parkreldis.2014.09.003)
Supplement: Supplementary file 1 [file mmc1.ppt]

## Slide 1
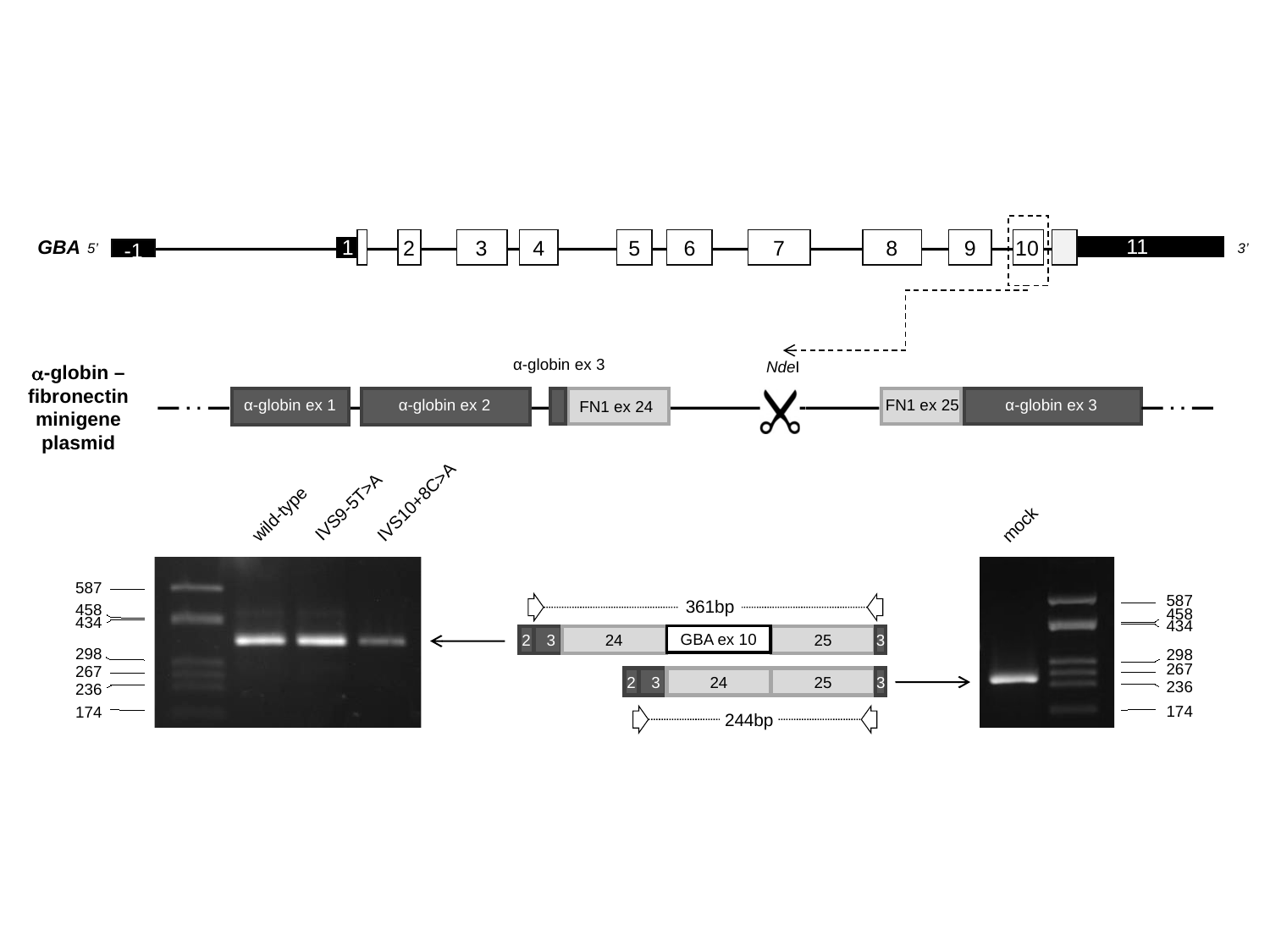

11
1
2
3
4
5
6
7
8
10
9
GBA
-1
5’
3’
α-globin ex 3
NdeI
α-globin ex 3
α-globin ex 1
α-globin ex 2
FN1 ex 25
FN1 ex 24
α-globin –fibronectin minigene plasmid
IVS10+8C>A
IVS9-5T>A
wild-type
mock
587
458
434
298
267
236
174
361bp
GBA ex 10
2
3
24
25
3
587
458
434
298
267
2
3
24
25
3
236
174
244bp
