# Supplementary material for: Glucocerebrosidase mutations in primary parkinsonism
Source: Parkinsonism Relat Disord. 2014 Nov;20(11):1215–20. doi: 10.1016/j.parkreldis.2014.09.003 (PMC4228056; doi:10.1016/j.parkreldis.2014.09.003)
Supplement: Supplementary file 2 [file mmc2.docx]

ONLINE SUPPLEMENTARY MATERIAL

**METHODS**

**Mutation analysis**

Genomic DNA extraction was performed from peripheral blood using a Microlab STAR Liquid Handler (Hamilton, Bonaduz, Switzerland) integrated with Chemagen automated DNA extraction system (Chemagen AG, Baesweiler, Germany). DNA samples were quantified using a Nanodrop spectrophotometer (NanoDrop Technologies, Wilmington, DE, USA), standardized for concentration (40 ng/µL for the source, 5 ng/µL for the working dilution) and arrayed into 96-deep-well plates.

PCR primer couples were designed on the basis of the known genomic sequence of the gene (GenBank accession number NM_000157) to amplify the two exons of interest and their exon-intron boundaries, avoiding the concomitant amplification of the highly-homologous GBA pseudogene (*GBAP1*) (Supplementary Table 1).

The mutational screening was hence performed by a combination of high-resolution melting (HRM) analysis (exon 9) and direct DNA sequencing (exon 10).

For HRM reactions, 7.5 ng of genomic DNA was amplified in a final volume of 10 µL. Reactions were performed in 384-well LightCycler 480 plates (Roche Applied Science, Indianapolis, IN, USA) using the LightCycler 480 HRM Master Mix (Roche). PCR cycling and HRM analysis were performed on a LightCycler 480 (Roche). Amplicons were analyzed with the Gene Scanning Software (Roche). The sensitivity and specificity of heterozygous single-base change detection were ensured by including, in each plate, the genomic DNA of a previously-identified heterozygous carrier of the p.N370S mutation as positive control [Srulijes et al., 2013]. All putative heterozygous individuals showing unusual HRM profiles were subjected to DNA sequencing for mutation confirmation/identification.

For DNA sequencing, standard PCRs were performed on 20 ng of genomic DNA in a 25-μL final volume using the FastStart Taq DNA Polymerase (Roche). Direct sequencing of PCR products was performed by the fluorescent dideoxy terminator method (BigDye Terminator Cycle Sequencing Ready Reaction Kit v1.1; Applied Biosystems, Foster City, CA, USA), and analyzed by using an ABI-3130XL Genetic Analyzer (Applied Biosystems). The Variant Reporter software was used for mutation detection (Applied Biosystems).

**Molecular characterization of novel splicing variants**

To examine the effects of the IVS9-5T>A and IVS10+8C>A variants on *GBA* pre-mRNA splicing, appropriate minigene constructs were generated. To this aim, the hybrid alpha-globin-fibronectin minigene plasmid (pBS-KS), in which the alternatively-spliced extra-domain-B (EDB) exon of fibronectin had been removed to generate a site for the insertion of exons under study, was used [Baralle et al., 2003]. A 438-bp-long *GBA* fragment, comprising 248 bp of intron 9, exon 10, and 73 of intron 10, was PCR amplified from the relevant heterozygous patient. Oligonucleotides used for the amplification carried a *Nde*I restriction site in their 5’ ends (Supplementary table 1), in order to clone the products into the pBS-KS vector. The obtained wild-type (pBS-KS-wt) and mutant (pBS-KS-IVS9-5T>A and pBS-KS-IVS10+8C>A) plasmids were isolated by the PureYield Plasmid Miniprep System (Promega, Madison, WI, USA). The correct orientation of the insert, as well as the presence/absence of the putative splicing variants, were verified by DNA sequencing.

Human cervical carcinoma HeLa cells were cultured according to standard procedures. Cells were transiently transfected with 4 μg of either the wild-type or the mutant vector using the FuGENE HD Transfection Reagent (Promega). Twenty-four hours after the transfection, total RNA was extracted using the Eurozol reagent (Euroclone, Wetherby, UK). One microgram of total RNA was reverse transcribed using random nonamers and the Superscript-III Reverse Transcriptase (Invitrogen, Carlsbad, CA, USA). Of a total of 20 µL, 1 µL was used as template for standard PCR reactions by means of primers mapping in the flanking fibronectin exonic regions of the plasmid (Supplementary table 1).

**Supplementary References**

Baralle M, Baralle D, De Conti L, Mattocks C, Whittaker J, Knezevich A, Ffrench-Constant C, Baralle FE. Identification of a mutation that perturbs NF1 agene splicing using genomic DNA samples and a minigene assay. J Med Genet. 2003;40:220-2.

Srulijes K, Hauser AK, Guella I, Asselta R, Brockmann K, Schulte C, Soldà G, Cilia R, Maetzler W, Schols L, Wenning GK, Poewe W, Barone P, Wüllner U, Oertel W, Berg D, Goldwurm S, Gasser T. No association of *GBA* mutations and multiple system atrophy. Eur J Neurol. 2013;20:e61-2. doi: 10.1111/ene.12086.

**Supplementary table 1: Primer couples used for *GBA* exons 9 and 10 screening, cloning, and RT-PCR assays.**

| ***Primer*** | ***Sequence (5’-3’)*** | ***Localization ^a^*** | ***Application*** |
| --- | --- | --- | --- |
| GBA_Int8_F2  GBA_Ex9_R | tgtgacccttacctacactctct  GACAAAGTTACGCACCCAAT | Intron 8, chr1:155,205,687-155,205,709  Exon 9, chr1:155,205,549-155,205,568 | *GBA* exon 9 mutational screening through HRM analysis |
| GBA_Int8_F1  GBA_Ex9_R | atgcctggatcttcacacc  GACAAAGTTACGCACCCAAT | Intron 8, chr1:155,205,779-155,205,797  Exon 9, chr1:155,205,549-155,205,568 | Identification/confirmation of exon 9 variants disclosed through HRM analysis |
| GBA_Ex9_F  GBA_Int10_R | ATTGGGTGCGTAACTTTGTC  tagggagcagggaggaga | Exon 9, chr1:155,205,549-155,205,568  Intron 10, chr1:155,204,895-155,204,912 | *GBA* exon 9 mutational screening through Sanger sequencing |
| IVS9_*Nde*I_F  IVS10_*Nde*I_R | GGAATTCCATATGcacacccatccccttttgca  GGAATTCCATATGctgagagtgtgatcctgcca | Intron 9, chr1:155,205,331-155,205,350  Intron 10, chr1:155,204,913-155,204,932 | Cloning of a *GBA* fragment (introns 9-10) for the molecular characterization of the IVS9-5T>A and IVS10+8C>A variants |
| GBA_EX7_F  GBA_EX11_R | AGTGGATACCCCTTCCAGT  AAGAGGCACATCCTTAGAGGAG | Exon 7, chr1:155,207,272-155,207,290  Exon 11, chr1:155,204,870-155,204,891 | Splicing assays (in-vivo experiment; IVS10+1G>T variant) |
| GBA_Ex8_FAM_F  GBA_EX11_R | FAM-CCAAGTTCTGGGAGCAGAGT ^b^  AAGAGGCACATCCTTAGAGGAG | Exon 8, chr1:155,206,090-155,206,109  Exon 11, chr1:155,204,870-155,204,891 | Fluorescent splicing assays (in-vivo experiment; IVS10+1G>T variant) |
| HBA1_2/3_F  FN1_25_R | CAACTTCAAGCTCCTAAGCCACTGC  TAGGATCCGGTCACCAGGAAGTTGGTTAAATCA | / | Minigene splicing assays (in-vitro experiments; IVS9-5T>A and IVS10+8C>A variants) |

Intronic sequences are in lower-case letters, exonic sequences are in upper-case letters; upper-case underlined sequences were introduced to allow the *Nde*I digestion in cloning procedures (5’-CATATG-3’, *Nde*I restriction site).

***^a^*** According to UCSC Genome Browser (http://genome.ucsc.edu/, release Feb. 2009; GRCh37/hg19 assembly).

^b^ Fluorescently labelled with the fluorophore 6-Fam (6-fluorescein amidite).

**Supplementary table 2. Association of *GBA* mutations in patients and controls.**

| ***Mutation/s*** | ***N. patient***  ***carriers (%)*** | ***N. control***  ***carriers (%)*** | ***OR (95% CI)*** | ***P value**** |
| --- | --- | --- | --- | --- |
| p.N370S | 69 (2.5%) | 4 (0.36%) | 7.1 (2.6-19.5) | 1.2 * 10^-6^ |
| p.L444P (all)  p.L444P (simple)  p.L444P (rec) | 47 (1.7%)  30 (1.1%)  17 (0.6%) | 3 (0.27%)  1 (0.09%)  2 (0.18%) | 6.4 (2.0-20.6)  12.1 (1.6-88.9)  3.4 (0.79-14.8) | 1.1 * 10^-4^  5.3 * 10^-4^  0.1237 |
| p.N370S  p.D443N  p.L444P  IVS10+1G>T | 118 (4.3%) | 7 (0.63%) | 7.03 (3.27 – 15.13) | 6.4 * 10^-11^ |

Test performed on 2,766 patients with degenerative parkinsonism and 1,111 controls screened for both exon 9 and exon 10.

The p.L444P mutation carriers were compared also distinguishing between carriers of the “sole” p.L444P mutation (simple) and carriers of the complex p.L444P+ p.A456P variant (indicating the presence of a complex recombinant allele; rec).

No OR was calculated for the two mutations being present only in 1 case and in none of the examined controls.

*******Statistics: Fisher exact test, two-tail.

In gray: all the pathogenic mutations considered together (see functional analysis for the splicing mutations); the E388K variant was excluded, being probably a rare polymorphism (in our study population, difference between case and control carrier frequency P=0.68).

**Legend to Supplementary figure 1: *In-vitro* analysis of the effect of the IVS9-5T>A and IVS10+8C>A genetic variants on *GBA* pre-mRNA splicing.**

A *GBA* region of 438 bp, containing exon 10 along with intronic flanking sequences, was PCR amplified from the genomic DNA of the two heterozygous carriers, and cloned into the α-globin-fibronectin minigene plasmid. We hence generated three recombinant plasmids: one showing the wild-type *GBA* sequence, and the others having either the IVS9-5T>A or the IVS10+8C>A mutation. Minigene constructs were transiently transfected into HeLa cells. Analysis of transcripts generated from the different allelic variants showed that in all cases exon 10 is correctly included into the mature mRNA, demonstrating that both IVS9-5T>A and IVS10+8C>A do not affect splicing.

In the upper part of the figure, a schematic representation of the *GBA* gene is reported: exons are represented by boxes (the thinner ones corresponding to the 5’ and 3’ untranslated regions) and introns by lines. The gene is approximately drawn to scale.

In the middle, a scheme of the hybrid pBS-KS-Exon10 minigene is shown: α-globin and fibronectin (*FN1*) exons are represented by gray boxes, whereas introns are represented by lines (not to scale). *GBA* exon 10 with its flanking intronic regions was cloned into the *Nde*I site of the pBS-KS_modified vector.

In the lower part of the figure, the results of RT-PCR experiments are shown. Amplified products were obtained from RNA of HeLa cells transfected with the empty vector (negative control, mock; right panel), the wild-type, or the mutant (IVS9-5T>A and IVS10+8C>A; left panel) minigene constructs, and separated on a 2% agarose gel. A schematic representation of the two obtained RT-PCR products is also reported. Primers used in RT-PCR experiments are indicated by arrows. The length of the fragments is also indicated.

**Case Descriptions**

1) *GBA*-N370S + *LRRK2*-G2019S

This 68-year-old man resulted to be a heterozygous carrier of the *LRRK2*-G2019S mutation and the *GBA*-N370S mutation. Clinical onset of Parkinson’s disease (PD) was in 1994 at 49 years of age with micrographia and resting tremor on the right lower limb. Levodopa therapy was immediately started with excellent motor response. He started complaining about motor fluctuations after 16 years from the onset, the association of pramipexole and entacapone greatly improved his wearing-off symptoms, while quetiapine reduced his night time restlessness. At that time, he developed impulse control disorders (such as pathological gambling and hypersexuality), which recovered after pramipexole dose reduction and psychotherapy. After one year, he developed visual hallucinations, whose insight was preserved. At his last examination at our institute on 2013 (68 years), he had severe on-off motor fluctuations and troublesome dyskinesia. His activity of daily living is reduced (UPDRS II: 14) and UPDRS motor scores were 24 (On-meds) and 40 (Off-meds). Brain MRI was unremarkable.

Cognitive and behavioural assessment was conducted twice (at 17- and 18-year disease duration, respectively) and, despite the long disease duration, he did not show any remarkable sign of cognitive dysfunction at both evaluations. Behavioural assessment disclosed his past history of ICD (recovered at follow-up) and current apathy and dysphoria.

He has no family history of movement disorders. Out of 3 siblings, one had Alzheimer’s dementia, with onset at 71 years of age.

2) *GBA*-N370S + *LRRK2*-G2019S

This 63-year old woman is heterozygous carrier of the *LRRK2*-G2019S mutation and the *GBA*-N370S mutation. PD motor symptoms started in 2002 when she was 52 with right leg clumsiness. A couple of years before she started complaining about depressive symptoms. A Dopamine Transporter SPECT scan (DATSCAN) confirmed reduced nigral neuronal density projecting to the left putamen. She was prescribed pramipexole up to 2.1 mg daily with partial recover of motor symptoms. Levodopa therapy was then initiated with excellent motor response. At 5-year disease duration brain MRI was normal and levodopa acute challenge at 200mg was positive with UPDRS motor score of 69% (OFF-state UPDRS part III = 13; ON score = 4). Motor symptoms progressively worsened over time with the appearance of a poor levodopa responsive freezing of gait, causing unbalance with sporadic falls. On 2013 (63-year old and 11-year disease duration), she was admitted to hospital for clinical and instrumental investigations. Her neurological examination revealed mild to moderate bradykinesia and muscle rigidity on the left body side, freezing of gait, unsteadiness at the pull-test in the OFF state. In the ON state she had moderate choreic movements at neck, trunk, upper limbs, but no freezing of gait. She underwent brain MRI (unremarkable) and a 150mg levodopa acute challenge (positive: 48% improvement of UPDRS part III scores, from 23 to 12). Neuropsychological assessment revealed normal functions in all domains (MMSE 30/30, Frontal Assessment Battery 17/18), while the behavioural assessment revealed the presence of impulse control disorder (pathological gambling) and moderate depressive symptoms. At that time, she was on Levodopa/Carbidopa/Entacapone 150mg four times daily and prolonged-release pramipexole 1.05mg.

She has no family history of movement disorders. Her medical history includes mitral and aortic valvulopathy, hypertension, transient ischemic attack in 1995.

3) *GBA*-N370S + *LRRK2*-R1441C

This patient was a 70-year-old man, carrier of *LRRK2*-R1441C and *GBA*-N370S mutations. PD presented when he was 44 with micrographia and right-sided bradykinesia. After 1 year, he started levodopa therapy with good response. After 3 years from the initiation of levodopa, he developed wearing-off and, subsequently, dyskinesias. At 14-year disease duration, he was finally referred for deep brain stimulation on the internal globus pallidus for severe on-off motor fluctuations, with good outcome.

At 62 years he presented sign of mild cognitive impairment. Full-blown dementia was diagnosed 5 years later. He died at age 70 of pneumonia.

Cognitive and behavioural assessment was conducted six times, the first at the age of 59 (one year after DBS surgery) and the last at 69 (disease duration 15 and 27 years, respectively). The first assessment revealed overall preserved cognitive functions. Three years later, he showed memory and frontal-lobe dysfunction, visual hallucinations, anxiety and sleep problems. One year later, memory and frontal-lobe functions were severely impaired with further worsening of neuropsychiatric symptoms (hallucinations, anxiety, and irritability). Finally, his severe dementia was associated with apathy, auditory and visual well-formed hallucinations along with delusions. Family history: patient was not available for a formal session of genetic counselling. He reported that his father had died at 87 years of age, with a formal diagnosis of PD at 80 years. No other relatives were reported to have had movement disorders or other neurodegenerative diseases.

4) *GBA* (N370S) + Parkin

This patient is a heterozygous carrier of the *GBA*-N370S mutation and carrier of an homozygous exon 3 deletion in the Parkin gene.

In this 48-year-old male patient, PD onset was in 2000 at the age of 37 years with left-sided slowness and muscles rigidity. He was diagnosed 2 years later and started levodopa and pramipexole with excellent response. Motor fluctuations started in 2008 with wearing off (characterized by gait disturbance and depressive symptoms), followed by mild trunk dyskinesias. At the last examination in 2011 (48 years, 11-year disease duration) the main motor problem in the OFF was gait, mainly because of left lower limb bradykinesia and rigidity, but postural reflexes remained normal. His Hoehn and Yahr stage was 2/5. In the ON-state, his UPDRS motor score improved from 25 to 6, with resolution of any motor symptom, including gait impairment. He had symptomatic orthostatic hypotension (95/70mmHg after 100mg levodopa). Peak-dose dyskinesias appeared only at levodopa dosage above 150mg. He had no sign of cognitive dysfunction, but had mild depressive symptoms.

In his family history, there is a sister, not available to our analysis, who developed PD at 38 years of age.
